# Modulatory Effect of Nicotinamide Adenine Dinucleotide Phosphate (NADPH) on the 2-Oxoglutarate Mitochondrial Carrier

**DOI:** 10.3390/molecules29215154

**Published:** 2024-10-31

**Authors:** Anna Spagnoletta, Daniela Valeria Miniero, Nicola Gambacorta, Francesca Oppedisano, Anna De Grassi, Orazio Nicolotti, Ciro Leonardo Pierri, Annalisa De Palma

**Affiliations:** 1 Laboratory “Regenerative Circular Bioeconomy”, ENEA-Trisaia Research Centre, 75026 Rotondella, Italy; 2 Department of Biosciences, Biotechnologies and Environment, University of Bari Aldo Moro, 70125 Bari, Italy; anna.degrassi@uniba.it (A.D.G.); annalisa.depalma@uniba.it (A.D.P.); 3 Department of Medicine & Surgery, LUM University Giuseppe Degennaro Torre Rossi, Piano 5 S.S. 100 Km. 18, 70010 Casamassima, Italy; 4 Department of Pharmacy-Pharmaceutical Sciences, University of Bari Aldo Moro, 70125 Bari, Italy; nicola.gambacorta1@uniba.it (N.G.); orazio.nicolotti@uniba.it (O.N.); 5 Department of Health Sciences, Institute of Research for Food Safety and Health (IRC-FSH), University “Magna Graecia” of Catanzaro, 88100 Catanzaro, Italy; oppedisanof@libero.it

**Keywords:** mitochondrial transport, oxoglutarate carrier, NADPH regulation, molecular docking, kinetic analysis, isocitrate/oxoglutarate shuttle, malate/aspartate shuttle, mitochondrial function

## Abstract

The 2-oxoglutarate carrier (OGC), pivotal in cellular metabolism, facilitates the exchange of key metabolites between mitochondria and cytosol. This study explores the influence of NADPH on OGC transport activity using proteoliposomes. Experimental data revealed the ability of NADPH to modulate the OGC activity, with a significant increase of 60% at 0.010 mM. Kinetic analysis showed increased Vmax and a reduction in Km for 2-oxoglutarate, suggesting a direct regulatory role. Molecular docking pointed to a specific interaction between NADPH and cytosolic loops of OGC, involving key residues such as K206 and K122. This modulation was unique in mammalian OGC, as no similar effect was observed in a plant OGC structurally/functionally related mitochondrial carrier. These findings propose OGC as a responsive sensor for the mitochondrial redox state, coordinating with the malate/aspartate and isocitrate/oxoglutarate shuttles to maintain redox balance. The results underscore the potential role of OGC in redox homeostasis and its broader implications in cellular metabolism and oxidative stress responses.

## 1. Introduction

Reactive oxygen species (ROS) and reactive nitrogen species (RNS) are critical signaling molecules derived from aerobic metabolism and are particularly active in the mammalian brain [1]. Under normal conditions, ROS and RNS levels are tightly regulated, ensuring the support for cellular functions, like signaling and immune response [2]. However, excessive production of these species can lead to oxidative stress, which damages cellular components, including lipids, proteins, and DNA, and contributes to the onset of various diseases [3,4], including cancer [5], diabetes [6], metabolic disorders [7] cardiovascular disorders [8], and neurodegenerative diseases [9]. Antioxidants targeting specific molecular pathways have been identified as promising therapeutic strategies for mitigating these effects [10].

In cells, the mitochondrial oxidative phosphorylation system (OXPH) and enzymes like NADPH oxidases (NOX) and nitric oxide synthases (NOS) are major sources of ROS and RNS [11,12]. Remarkably, several NADPH-dependent enzymes, such as glucose-6-phosphate dehydrogenase (G6PDH) and 6-phosphogluconate dehydrogenase (6PGD) along the pentose pathway, are responsible for the cytosolic NADP^+^/NADPH turnover and contribute to maintaining cellular redox balance [13,14]. In addition, the NADP^+^-dependent malic enzymes (ME1 and ME3) contribute to cellular redox balance by regenerating reduced glutathione (GSH) and participating in various biosynthetic pathways [15,16]. Excessive ROS, particularly mitochondrial superoxide (O_2_^•−^), which is converted into reactive H_2_O_2_, is a major driver of oxidative stress-related pathologies like Parkinson’s and Alzheimer’s diseases [17]. Mitochondrial defense mechanisms, such as the glutathione redox cycle (GSH/GSSG) and mitochondrial glutathione peroxidase (GPX), provide critical protection against ROS-induced damage [18]. The redox state, reflected by the NAD(P)^+^/NAD(P)H ratio, is crucial for maintaining metabolic flexibility and coordinating cytosolic and mitochondrial responses to oxidative stress. Several NADP^+^-dependent dehydrogenases, including the isocitrate dehydrogenase (IDH) enzyme and other NAD(P)^+^-dependent dehydrogenases, help to regulate the cellular NAD(P)^+^/NAD(P)H ratio, which is crucial for redox homeostasis and mitochondrial-cytosolic interactions [17,18,19,20]. Among the enzymes participating in the regulation of this balance, apoptosis-inducing factor (AIF) and dihydrolipoamide dehydrogenase (DLD) deserve to be mentioned for their key additional role in the regulation of mitochondrial apoptosis [21,22,23,24,25,26].

Among mitochondrial carriers, the mitochondrial 2-oxoglutarate carrier (OGC) is essential for energy metabolism and redox balance. Indeed, OGC facilitates the exchange of the mitochondrial 2-oxoglutarate for the cytosolic malate [23]. Remarkably, cytosolic oxoglutarate primarily originates from the tricarboxylic acid cycle (TCA) in the mitochondria, where it is generated by the oxidative decarboxylation of isocitrate by isocitrate dehydrogenase (IDH). It is then transported out of the mitochondria by OGC [23], contributing to the malate/aspartate shuttle [27,28,29,30,31,32,33] that ensures the proper transfer of reducing equivalents from NADH and NADPH between the cytosol and mitochondria [23,34]. It has been studied for its role in maintaining redox homeostasis in tissues such as the brain and liver, and for its interactions with hemin, which influence cellular oxidative balance [35,36,37,38]. Moreover, 2-oxoglutarate also serves as a key intermediate in various biosynthetic pathways and redox reactions in cytosol, including amino acid synthesis and nitrogen metabolism. In addition to its mitochondrial origin via the TCA cycle, 2-oxoglutarate can also be produced in cytosol by cytosolic glutamate dehydrogenase (GDH) and aminotransferases, as well as by the action of cytosolic isocitrate dehydrogenase (IDH1), which converts isocitrate to oxoglutarate and plays a critical role in NADPH generation and redox regulation [15,19,23].

Although OGC plays a key role in redox processes, studies have shown that it does not directly transport glutathione (GSH), which is central to oxidative stress regulation [39,40,41,42]. Nevertheless, impaired OGC function has been linked to reduced energy production and increased oxidative stress, contributing to neurological and hepatic disorders [43,44,45]. Recent findings have revealed the broader regulatory role of NADPH in redox biology. NADPH not only serves as a cofactor in biosynthetic reactions but also acts as a critical redox sensor that helps regenerate antioxidants like glutathione and thioredoxin. These processes are vital in protecting cells from oxidative damage, especially in organs with high metabolic activity such as the brain, where redox imbalances can lead to pathologies like ischemia-reperfusion injury and neurodegeneration [15,19]. Despite the importance of NADPH in redox systems, its direct interaction with mitochondrial carriers like OGC has not been extensively studied. This study seeks to address this gap by investigating the modulatory effect of NADPH on OGC transport activity. Our data show that NADPH increases OGC activity by 60%, suggesting a potential regulatory mechanism that enhances mitochondrial function under oxidative stress conditions. This modulation could position OGC as a key player in coordinating mitochondrial redox states with broader metabolic pathways such as the isocitrate/oxoglutarate and malate/aspartate shuttles [37,38,39]. Understanding the interaction between NADPH and OGC has implications beyond neurodegenerative diseases. Given NADPH’s key role in antioxidant defense and anabolic processes, targeting its interaction with OGC could help combat oxidative stress in conditions like cancer, diabetes, and cardiovascular diseases. Clarifying the mechanisms behind OGC activity may lead to new therapies that modulate mitochondrial function and oxidative balance, offering potential treatments for disorders linked to mitochondrial dysfunction. This study’s findings suggest that targeting mitochondrial carriers like OGC could play a crucial role in regulating NADPH-dependent redox homeostasis and reducing oxidative damage in such conditions.

## 2. Results

### 2.1. Transport Activity of Reconstituted OGC in the Presence of GSH, GSSG, NADPH and NADP^+^

Firstly, the substrate specificity of OGC towards GSH, GSSG, NADP^+^, and NADPH, which are involved in redox homeostasis, was investigated to evaluate their potential role as substrates. These molecules were loaded into liposomes reconstituted with OGC protein (0.02 µg/µL final concentration in proteoliposomes), and the uptake of 2-oxoglutarate was measured. According to Ref. [46], the results reported in Table 1 clearly show that the highest transport rate was obtained with the physiological substrate malate (3.385 ± 120 nmol 10 min⁻^1^ mg⁻^1^ protein). Conversely, the reconstituted OGC did not catalyze the hetero-exchange of external 2-oxoglutarate for internal GSH, GSSG, NADP^+^, or NADPH. Therefore, the residual activity of OGC in the presence of these compounds was approximately the same as in the presence of NaCl or boiled OGC prior to incorporation into liposomes These results ruled out the possibility of these compounds serving as OGC substrates, confirming findings from the literature [39,41,47].

When GSH, GSSG, NADP^+^, and NADPH (each at a concentration of 2 mM) were added as inhibitors outside of OGC proteoliposomes together with 0.1 mM [^14^C] 2-oxoglutarate, the malate/2-oxoglutarate transport activity was reduced by 10% in the presence of GSH and significantly increased by 24% with NADPH, while no effects were observed with NADP^+^, GSSG, or NaCl (Table 2).

### 2.2. Effect of GSH and NADPH on OGC Transport Activity

**Figure 1:**
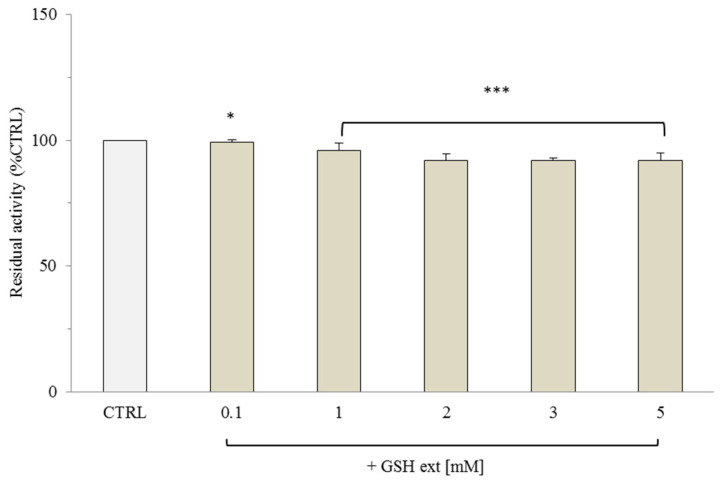
Transport activity of [^14^C] 2-oxoglutarate in the reconstituted OGC in the presence of external GSH (GSH ext). The OGC proteoliposomes were preloaded with 10 mM of malate, and different concentrations of GSH (0.1–5 mM) were incubated for 3 min. Transport was started by adding 0.1 mM of [^14^C] 2-oxoglutarate and stopped after 10 min with 350 mM PLP. The control (CTRL) was represented by proteoliposomes in the absence of external GSH, replaced by the buffer (50 mM NaCl,10 mM Pipes, pH 7). The results were the average of at least three experiments ± S.D. Statistical analysis was performed using one-way ANOVA followed by Dunnett’s post-hoc test for multiple comparisons. Indicate * *p* < 0.05, *** *p* < 0.001 compared to the control group.

Since GSH and NADPH slightly modify OGC transport activity when added externally to proteoliposomes, we further investigated the mild inhibitory effect of GSH and the more significant activating effect of NADPH using a concentration range similar to physiological levels. Considering mitochondrial GSH concentrations (1–5 mM) [15,48], the uptake of labeled 2-oxoglutarate mediated by purified and reconstituted OGC was studied in proteoliposomes internally loaded with 10 mM malate and increasing concentrations of GSH, ranging from 0.1 mM to 5 mM. As shown in Figure 1, OGC transport activity was not significantly affected by GSH across the entire range of tested concentrations, excluding any major role of GSH in modulating the OGC transport rate.

**Figure 2:**
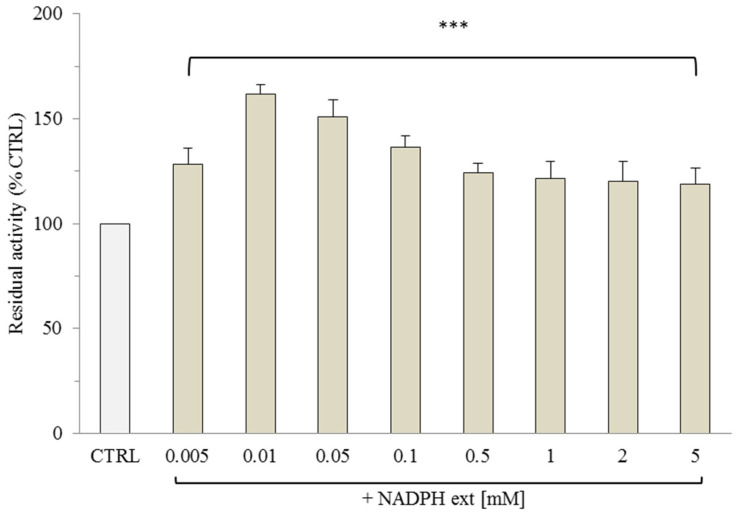
Transport activity of [^14^C] 2-oxoglutarate in the reconstituted OGC in the presence of external NADPH (NADPH ext). The OGC proteoliposomes were loaded with 10 mM of malate, and different concentrations of NADPH (0.005 mM–5 mM) were incubated for 3 min. Transport was started by adding 0.1 mM of [^14^C] 2-oxoglutarate and stopped after 10 min with 350 mM of PLP. The control (CTRL) was represented by proteoliposomes incubated without external NADPH replaced in CTRL assays by the buffer (50 mM NaCl, 10 mM Pipes, pH 7). The results were the average of at least three experiments ± S.D. All groups showed a statistical significance of *** *p* < 0.001 compared to the control, as determined by one-way ANOVA and Dunnett’s post-hoc test.

Similarly, the increase in OGC transport activity induced by NADPH was studied to understand the possible interaction between OGC and this substrate, which is involved in many ROS scavenging mechanisms. Considering the physiological concentration of NADPH in mammalian mitochondria (0.01–0.1 mM) [12], the uptake of 0.1 mM [^14^C] 2-oxoglutarate mediated by reconstituted OGC in malate-loaded proteoliposomes was measured in the presence of various concentrations of NADPH (0.005–5 mM). The results of 2-oxoglutarate/malate exchange, shown in Figure 2, indicate that transport activity increased significantly at very low concentrations (0.005–0.01 mM) and gradually decreased at higher concentrations (0.5–5 mM), stabilizing at levels higher than the control. This “modulatory effect” exerted by NADPH may suggest a potential direct interaction between OGC and NADPH.

**Figure 3:**
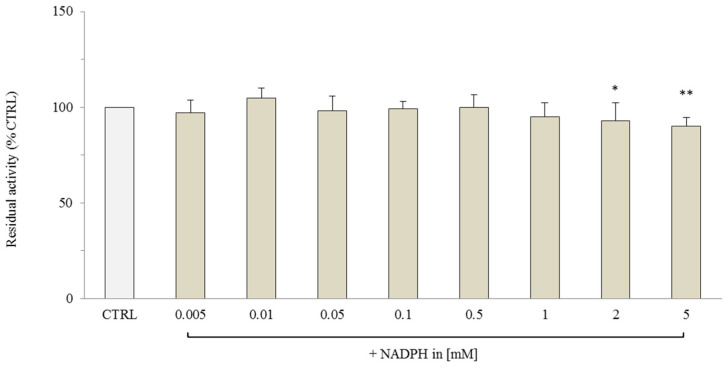
Transport activity of [^14^C] 2-oxoglutarate in the reconstituted OGC in the presence of internal NADPH (NADPH in). The OGC proteoliposomes were loaded with 10 mM of malate and different concentrations of NADPH (0.005–5 mM). Transport was started by adding 0.1 mM of [^14^C] 2-oxoglutarate and stopped after 10 min by 350 mM of PLP. The control (CTRL) was represented by proteoliposomes loaded with only malate 10 mM. The results are the average of three experiments ± S.D. Statistical analysis was performed using one-way ANOVA followed by Dunnett’s post-hoc test for multiple comparisons. Indicate * *p* < 0.05, ** *p* < 0.01 compared to the control group.

Furthermore, to verify whether the effect of NADPH on the OGC carrier was also present within the proteoliposomes, we analyzed the uptake of 0.1 mM [^14^C] 2-oxoglutarate over 10 min in proteoliposomes loaded with NADPH at the same concentration range (0.005–5 mM) along with 10 mM malate. The data in Figure 3 show that no NADPH effect on the malate/2-oxoglutarate exchange was detected, suggesting that internal NADPH did not significantly modulate OGC transport activity.

**Figure 4:**
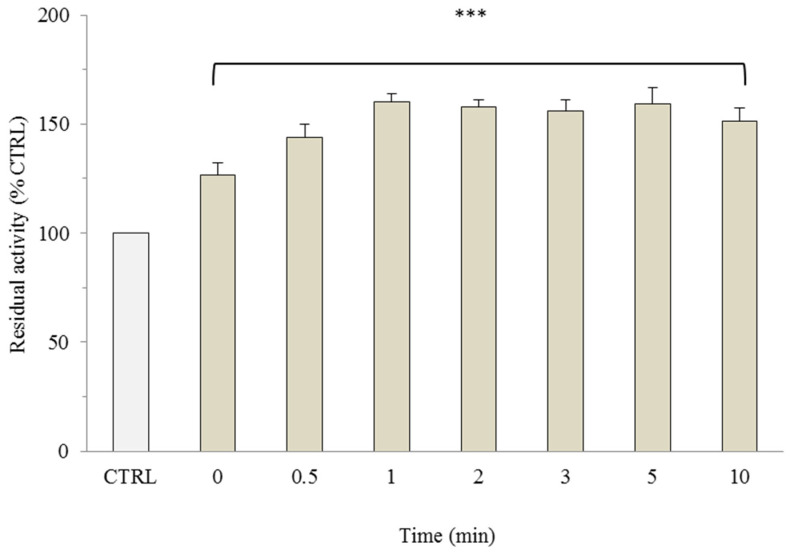
Dependence of external NADPH incubation time on OGC transport activity. OGC proteoliposomes were preloaded with 10 mM of malate and incubated with 0.01 mM of NADPH at different times. The exchange was started by adding 0.1 mM [^14^C] 2-oxoglutarate and stopped after 10 min with 350 mM of PLP. The control (CTRL) was represented by proteoliposomes incubated without external NADPH and in the presence of only buffer 50 mM NaCl, 10 mM Pipes, pH 7. The results are the means of three experiments ± S.D. All groups showed a statistical significance of *** *p* < 0.001 compared to the control, as determined by one-way ANOVA and Dunnett’s post-hoc test.

Additionally, the effects of oxidized GSSG and NADP^+^ were evaluated at the same concentration range as their reduced forms (i.e., 0.1–5 mM and 0.005–5 mM, respectively), and no modulation was observed Moreover, the stimulation of OGC transport activity was further investigated by varying the incubation time with 0.01 mM NADPH, the concentration that resulted in the most significant increase. Figure 4 shows that incubation of proteoliposomes with 0.01 mM NADPH for 1 min led to a time-dependent accumulation of radiolabeled 2-oxoglutarate, reaching up to 60% compared to the control, and remained constant with longer incubation times. In another set of experiments, OGC directly eluted from a chromatographic purification column was incubated with 0.01 mM NADPH for the same times, and after reconstitution, the uptake of 0.1 mM radiolabeled 2-oxoglutarate was measured In this case, no effect was observed, indicating that the modulatory effect exerted by external NADPH was likely due to a specific interaction in a region of the refolded OGC.

### 2.3. Kinetic Analysis of the Malate/2-Oxoglutarate Transport Activity by NADPH Modulation 

**Figure 5:**
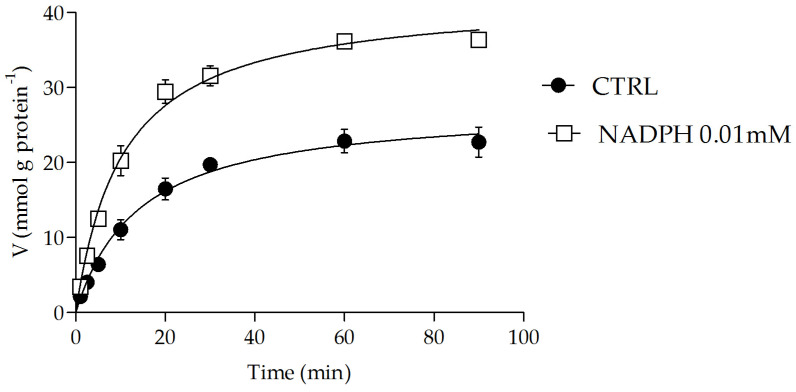
Time course of the exchange malate/2-oxoglutarate in reconstituted OGC in the presence of external NADPH. The time course was started by the addition of 0.1 mM of [^14^C] 2-oxoglutarate to proteoliposomes preloaded with 10 mM of malate in the absence (●) or in the presence of 0.01 mM (□). The transport was stopped at the indicated times by adding 350 mM of PLP, and the intraliposomal radioactivity was measured. The data are the means ± S.D. from three different experiments.

**Figure 6:**
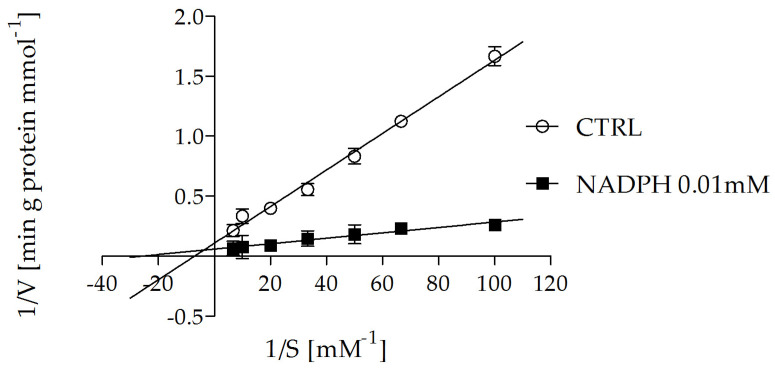
Lineweaver–Burk analysis of 2-oxoglutarate/malate exchange in the presence of external NADPH catalyzed by the reconstituted OGC. The uptake rate of [^14^C] 2-oxoglutarate in proteoliposomes containing 10 mM of malate was measured in 2 min in the absence (o) or the presence of 0.01 mM NADPH (■) incubated for 3 min. [^14^C] 2-oxoglutarate was added at the concentrations of 0.01 mM, 0.015 mM, 0.02 mM, 0.03 mM, 0.05 mM, 0.1 mM, and 0.15 mM. The proteoliposome control (CTRL) was incubated in the presence of only 50 mM NaCl, 10 mM Pipes 10 mM, pH 7, in the absence of external NADPH. The data are the means ± S.D. from three different experiments.

It was previously shown that NADPH significantly increased the uptake of labeled 2-oxoglutarate when incubated outside of proteoliposomes, and this activation was found to be time-dependent. To further investigate the influence of NADPH on the ability of reconstituted OGC to catalyze the transport of 2-oxoglutarate across proteoliposomes, a typical time-course experiment was performed, as shown in Figure 5. The uptake of [^14^C] 2-oxoglutarate into proteoliposomes loaded with unlabeled malate, in the absence and presence of 0.01 mM external NADPH, is reported as a function of time. It can be observed that in all cases, the 2-oxoglutarate uptake increased linearly for about 2 min, and the intraliposomal accumulation of 0.1 mM [^14^C] 2-oxoglutarate at approximately 90 min was significantly higher in the co-presence of 0.01 mM NADPH, as indicated by the following values: 36.1 mmol 90 min⁻^1^ g⁻^1^ protein in the presence of 0.01 mM NADPH and 23.0 mmol 90 min⁻^1^ g⁻^1^ protein in its absence. The exchange reactions followed first-order kinetics, with the first-order rate constants (k) for the 2-oxoglutarate/malate exchange being 0.0645 min⁻^1^ in the absence of NADPH and 0.0828 min⁻^1^ in the presence of 0.01 mM NADPH. Furthermore, the 2-oxoglutarate/malate exchange was studied using seven different concentrations of external [^14^C] 2-oxoglutarate in proteoliposomes loaded with 10 mM malate, both in the absence and presence of 0.01 mM NADPH. The activity data are shown in Figure 6 as a Lineweaver–Burk plot of external substrate concentrations. Straight lines were obtained that tended to converge at a common point in the second quadrant of the Cartesian plane. The maximum transport rate (Vmax) increased from 7.5 ± 0.9 mmol g⁻^1^ protein min⁻^1^ in the absence of NADPH to 20.1 ± 1.2 mmol g⁻^1^ protein min⁻^1^ in the presence of 0.01 mM NADPH. Similarly, the Km decreased from 0.11 ± 0.04 mM to 0.05 ± 0.001 mM, supporting the hypothesis that NADPH increases the affinity of OGC for the substrate, likely by binding to a region distinct from the binding site.

**Figure 7:**
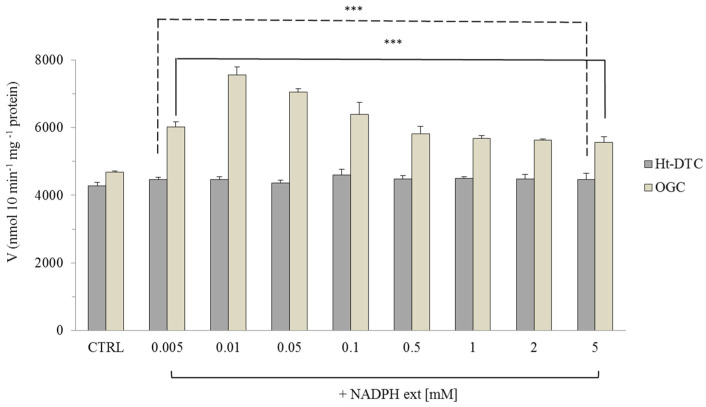
Transport activity of [^14^C] 2-oxoglutarate in the reconstituted *Ht*-DTC (dicarboxylate-tricarboxylate carrier) from *Heliantus tuberosus* and OGC (2-oxoglutarate carrier) from rat brain in the presence of external NADPH (NAPDH ext). Different concentrations (0.005–5 mM) of NADPH were added externally on OGC and *Ht*-DTC proteoliposomes preloaded with 10 mM of malate and incubated for 3 min. The transport was started by adding 0.1 mM [^14^C] 2-oxoglutarate and stopped after 10 min by 350 mM of PLP. The proteoliposome control (CTRL) was incubated in only 50 mM NaCl, 10 mM Pipes, pH 7 without NADPH. The data are the means ± S.D. from three different experiments. All groups showed a statistical significance of *** *p* < 0.001 compared to their control, as determined by one-way ANOVA and Dunnett’s post-hoc test. The dashed and solid lines refer to the *Ht*-DTC and OGC data respectively.

Finally, to determine whether NADPH modulation was unique to the animal kingdom, the effect of NADPH on the uptake of 0.1 mM [^14^C] 2-oxoglutarate into proteoliposomes reconstituted with purified dicarboxylate-tricarboxylate carrier (*Ht*DTC) from *Helianthus tuberosus* mitochondria, the closest plant homolog to OGC, was evaluated [49]. The results clearly indicated that NADPH, when added externally to proteoliposomes containing *Ht*DTC (Figure 7), did not exert any modulatory effect, confirming that the specific interaction is limited to the mammalian OGC.

### 2.4. Molecular Docking Analyses

**Figure 8:**
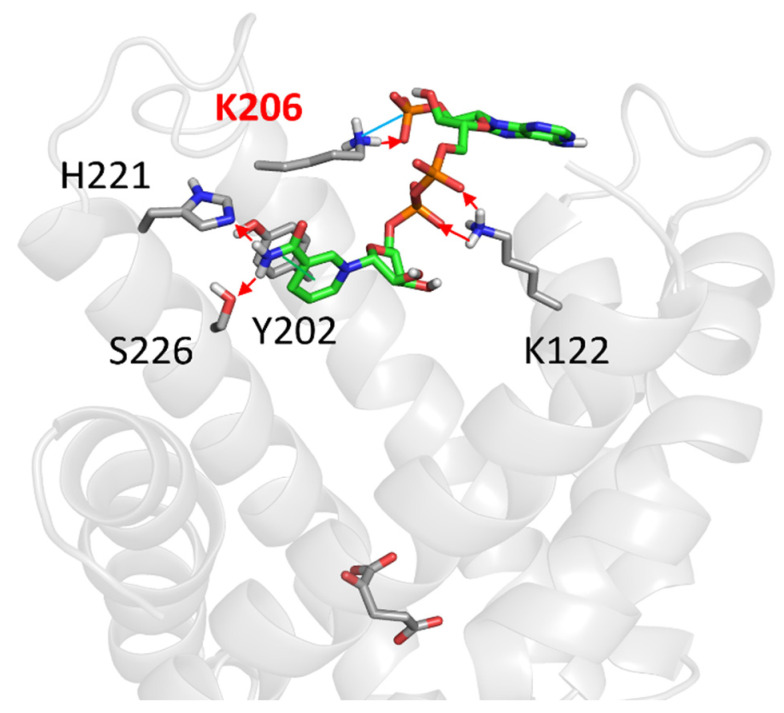
The panel reported the molecular docking analysis for NADPH (green sticks) within a putative cytosol-facing binding site. Red arrows and cyan and green lines showed the hydrogen bonds, electrostatic and π–π interactions, respectively. Blue, orange, white, and red colored atoms indicate Nitrogen, Phosphorous, Hydrogen, and Oxygen atoms. Carbon atoms are reported in light grey or green in protein residues and cofactor, respectively.

Finally, a molecular docking analysis was conducted to explore the possibility of a direct interaction between NADPH and specific regions of OGC, providing insights into NADPH’s interaction with the carrier. The analysis focused on the cytosol-facing region, including the cytosolic loops between helices 2 and 3 (residues E107–T115) and helices 4 and 5 (residues K206–N218). These regions include two positively charged residues, K206 and R108, whose cysteine mutations were previously shown to slightly impair the carrier’s functionality [33]. The molecular docking results, shown in Figure 8, revealed that NADPH could form interactions with the positively charged residues K206 and K122 through a strong hydrogen bond network via its phosphate moieties. Additionally, the nicotinamide ring of NADPH was observed to form hydrogen bonds with H221 and S226, and an aromatic interaction with Y202. The docking analysis yielded a binding score of −7.71 kcal/mol, supporting the feasibility of this interaction.

## 3. Discussion

In this study, we investigated the possible interactions between GSH, GSSG, NADPH, and NADP^+^, essential molecules in mitochondrial defense mechanisms against oxidative stress, and the OGC purified from rat brain mitochondria and reconstituted in liposomes by using measurements of transport activity to evaluate the potential involvement of this carrier in oxidative stress protection. None of these molecules were found to be translocated by OGC, consistent with previous reports [39,41]. However, a slight increase in OGC transport activity was observed in the presence of 2 mM NADPH outside the proteoliposomes (Table 1). To further investigate the potential modulation of OGC transport activity by NADPH, a wide range of NADPH concentrations (0.005–5 mM) was tested in OGC transport assays. It was observed (Figure 2) that NADPH (0.005–0.010 mM) increased OGC transport activity by up to 60% compared to the control. This result, along with the time-dependent effects of incubating NADPH at 0.010 mM (Figure 4), suggested a potential role for NADPH in modulating OGC activity. Kinetic investigations (Figure 6) revealed that the addition of 0.010 mM NADPH led to an increase in the transport rate (Vmax) from 7.5 ± 0.9 to 20.1 ± 1.2 mmol g^−1^ protein min^−1^ and a reduction in Km for 2-oxoglutarate from 0.11 ± 0.04 to 0.05 ± 0.001 mM compared to the control. Finally, molecular docking analysis was performed on the OGC cytosolic loops, where cysteine-scanning mutagenesis previously identified residues critical for OGC function [33]. Specifically, K206, located on the cytosolic loop between transmembrane helices H4 and H5, and R108, located on the loop between H2 and H3, were found to be essential, as their mutation to cysteine resulted in a 50% loss of OGC function. These residues are also adjacent to E107 and L209, mutations of which caused more than an 80% reduction in OGC function [33]. The docking analysis indicated a possible NADPH binding region on the cytosolic loops between helices H2 and H3 (residues E107–T115) and helices H4 and H5 (residues K206–N218), with selective interactions involving the phosphate groups of NADPH and residues K206 and K122, the latter located at the N-terminal portion of transmembrane helix H3. 

Based on this docking analysis, it is possible to suggest that NADPH interacts with the investigated OGC binding region. The identified binding site on the OGC cytosolic loops appears to act as a regulatory region, similar to those found on the matrix-facing side of other mitochondrial carrier family members [29,37,38,50,51,52]. 

**Figure 9:**
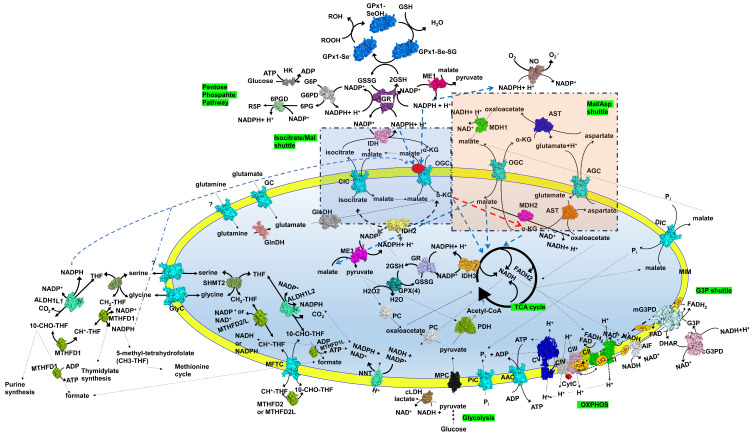
Scheme of a mitochondrion with a set of representative proteins, pathways and cycles. NADPH-OGC interactions participate in the regulation of cytoplasmic/mitochondrial NADPH-coupled redox systems. The above reported scheme indicates schematically a possible interaction of NADPH with OGC at the level of the isocitrate/2-oxoglutarate shuttle. According to the reported transport assays, in the presence of NADPH (0.01 mM), an increased activity of OGC is observed, depending on a possible interaction between NADPH (red sphere) and the OGC (in the cyan left box) at the level of OGC cytosolic loops (OGC reported in the cyan cartoon). Respiratory chain complexes, mitochondrial transporters, and other proteins are reported and labeled in surf representation. ATP synthase (CV) is reported in blue (based on the *Bos taurus* crystallized structure 6zqn.pdb). Mitochondrial carriers are reported in cyan (based on the 3D structure of the bovine ADP/ATP carrier, 1okc.pdb). MPC (an in-house developed 3D comparative model, data not published) is reported in black; PDH in light green (based on the human crystallized structure 6cfo.pdb); AIF in white (based on the human crystallized structure 4bur.pdb). Complex I (CI, based on the *Ovis aries* crystallized structure 5lnk.pdb), complex II (CII, based on the *Sus scrofa* scrofa 3aef.pdb), complex III (CIII, based on the *O. aries* 6q9e.pdb), and complex IV (CIV) (together with CytC in red, based on the bovine crystallized structure 5iy5.pdb) are reported in green, yellow, magenta, and grey, respectively. The human isocitrate dehydrogenase 2 (IDH2, pale yellow surface representation in the matrix, based on 6kdf.pdb), the human isocitrate dehydrogenase 3 (IDH3, orange surface representation in the matrix, based on 6kdf.pdb), the human isocitrate dehydrogenase 1 (IDH1, pink surface, in the cytosol, based on 1t09.pdb), the human mitochondrial glutamate dehydrogenase (GluDH, gray surface, in the matrix based on 1l1f.pdb), the human mitochondrial glutaminase (GLS, salmon surface, based on 6ujm.pdb), the human nicotinamide nucleotide transhydrogenase (NNT1, light green, inner mitochondrial membrane based on 1u31.pdb); the human cytosolic lactic dehydrogenase (LDH, yellow-sand color, based on 6baz.pdb); the human cytosolic (ME1) and matrix (ME3) malic enzyme (colored in brilliant pink and violet, respectively, based on 1pjl.pdb), the human glutathione reductase (GR, dark magenta surface in the cytosol and light magenta surface in the matrix, based on 1gre,pdb), the human selenocysteine to glycine glutathione peroxidase 4 (GPX4, petroleum blue surface, in the matrix, based on 2gs3.pdb), the human glucose-6-phosphate dehydrogenase (G6PD, grey surface, based on 5ukw.pdb), the human 6-phophogluconate dehydrogenase (6PGD, light green surface, based on 5uq9.pdb), the human hexokinase (HK, dark grey surface, based on 1hkc.pdb), the human c/m glycerol-3-phosphate dehydrogenase (m/c-G3PDH, cyan/pink surface, based on 2pla.pdb), the human cytosolic selenocysteine to glycine mutant of human glutathione peroxidase 1 (GPx1-Se-; Gpx1-SeOH; Gpx1-Se-SG, blue surface, in the cytosol based on 2f8a.pdb), NADH oxidase (NOX, dark salmon surface representation in the cytosol, based on 8x2l.pdb), the human serine hydroxy-methyltransferase (SHMT2, green smudge surface representation, based on 8tlc.pdb), the human methylenetetrahydrofolate dehydrogenase 2 (MTHFD2, green surface representation, based on 7ehj.pdb), and the human 10-formyltetrahydrofolate dehydrogenase (ALDH1L1, light green surface, based on 7rlu.pdb), according to PyMOL colors. Black circular arrows indicate cyclic pathways. Black solid/dashed lines indicate the possible direction of the reported reactions or metabolite fates. Blue dashed lines indicate reactions involving NADPH or OGC substrates (regarding malate or 2-oxoglutarate). The red dashed line indicates a possible asp/mal shuttle stimulation mediated by an increased uptake of 2-oxoglutarate. Other abbreviations: MIM: mitochondrial inner membrane; UQ, ubiquinone; AAC, ADP/ATP carrier, coded in *H. sapiens* by SLC25A4, SLC25A5, SLC25A6, SLC25A31; AGC, aspartate/glutamate carrier, coded by SLC25A12 and SLC25A13; DIC, dicarboxylate carrier, coded by SLC25A10; MFTC, assumed to be the FAD (folate/riboflavin) carrier, coded by SLC25A32; OGC, malate/2-oxoglutarate carrier, coded by SLC25A11; CIC, citrate carrier, coded by SLC25A1; PiC, phosphate carrier, coded by SLC25A3; MAS, malate/aspartate shuttle reported in an orange transparent box; TCA, tricarboxylic acid cycle; MDH1, cytosolic malate dehydrogenase 1; ME1, malic enzyme 1; MPC, mitochondrial pyruvate carrier; PDH, pyruvate dehydrogenase; CytC, cytochrome C; AIF, apoptosis-inducing factor. “?” indicate uncharacterized mitochondrial carriers.

Given these observations, we propose that OGC may function as a sensor responsive to changes in the redox state and cellular levels of NADPH, under both physiological and pathological conditions, as demonstrated for other mitochondrial transporters [13,29,53]. OGC could work in coordination with components of the malate/aspartate shuttle and the isocitrate/oxoglutarate shuttle [54]. In the context of the isocitrate/oxoglutarate shuttle, IDH2 is the primary source of NADPH, which is necessary for GSH regeneration in the mitochondria [55]. During physiological and “controlled” oxidative stress, where the balance between damage (ROS production) and repair (GSH/GSSG cycle) mechanisms is maintained, NADH and NADPH levels remain within cellular physiological ranges [56]. Under such conditions (0.01–0.1 mM NADPH) [15], we hypothesize that OGC primarily supplies the Krebs cycle with carbon skeletons [57]. However, as reported by Tretter and Adam-Vizi [17], an increase in H_2_O_2_ concentrations due to redox imbalance leads to a decline in mitochondrial NADPH and NADH levels. These authors also reported that mitochondrial concentrations of 50 µM H_2_O_2_ (indicating mild oxidative stress) induce a reversible inhibition of the aconitase enzyme, reducing 2-oxoglutarate production in the Krebs cycle and activating the enzyme bypass system involving aspartate aminotransferase. In this scenario, OGC would be activated in the presence of NADPH, with maximal activation occurring at 0.01 mM NADPH, ensuring a continuous supply of 2-oxoglutarate to the tricarboxylic acid cycle and maintaining NADH levels in the mitochondria. Conversely, higher concentrations of H_2_O_2_ lead to the irreversible inhibition of 2-oxoglutarate dehydrogenase (2-KGDH), a severe reduction in NADH, a blockade of the Krebs cycle, and eventually, cell death [17]. Our analysis suggests that low cytosolic concentrations of NADPH (0.01 mM) stimulate OGC exchange activity in the context of the isocitrate/2-oxoglutarate shuttle, leading to increased uptake of 2-oxoglutarate and stimulation of the Krebs cycle and the malate/aspartate shuttle. This effect may also influence cytoplasmic and mitochondrial NADP^+^/NADPH-dependent redox systems involved in GSH regeneration, purine biosynthesis, glycolysis, and the pentose phosphate pathway [13,15,16,53,58] (Figure 9). This modulatory effect of NADPH was not observed in the plant homolog of OGC-related mitochondrial transporters (e.g., DTC), likely due to the distinct defense mechanisms plants use to counteract ROS-induced damage and their broader array of NADP^+^/NADPH-dependent enzymes compared to animal cells [15].

## 4. Materials and Methods

### 4.1. Chemicals

[^14^C] 2-oxoglutarate was purchased from Perkin–Elmer Life Sciences; Hydroxylapatite (Bio-gel HTP) and Amberlite Bio-Beads SM-2 from Bio-Rad (Bio-Rad Laboratories S.r.l., Segrate (Mi), Italy); Matrex Gel Orange from Amicon (Beverly, MA, USA); acrylamide and N,N-methylenebisacrylamide from Serva (PRODOTTIGIANNI, Milan, Italy); Triton X-100, Triton X-114, egg yolk phospholipids from Fluka; NADPH, NADP^+^, GSH, GSSG, cardiolipin, 1,4-piperazine-diethanesulphonic acid (Pipes), sodium dodecyl sulfate (SDS) and asolectin from Sigma (Merk Life Science S.r.l., Milan, Italy); celite 535 from Roth and Sephadex G-75 from Pharmacia (Pharmacia & Upjohn Company, LLC, Peapack, NJ, USA) All other chemicals used were of analytical grade.

### 4.2. Purification of the OGC Carrier from Rat Brain Mitochondria

The 2-oxoglutarate carrier (OGC) was purified from rat brain mitochondria according to a protocol described in reported literature [46]. Briefly, mitochondria suspension was solubilized with a Buffer A containing 3% Triton X-100 (*w*/*v*), 20 mM Na_2_SO_4_, 1 mM EDTA, and 10 mM Pipes, pH 7.0 to a final protein concentration of 10 mg mL^−1^. After 10 min of incubation at 0 °C, the mixture was centrifuged at 15,000× *g* for 15 min. Then, the supernatant, supplemented with 4 mg mL^−1^ of cardiolipin, was applied to 0.6 g of cold hydroxyapatite/celite (5:1) columns and eluted in the presence of Buffer A. As previously reported, the first fraction (0.6 mL) was collected and applied to a cold Matrex Gel Orange A column, and pure OGC was isolated with an apparent molecular weight of 35 kDa [46]. All the operations were performed at 4 °C.

### 4.3. Purification of the DTC Carrier from Heliantus Tuberosus (HtDTC) Mitochondria

The DTC carrier was purified from *Heliantus tuberosus* mitochondria according to a protocol described in [49]. Briefly, mitochondrial suspensions were solubilized with a Buffer A containing 3% Triton X-100 (*w*/*v*), 20 mM Na_2_SO_4_, 1 mM EDTA, and 10 mM Pipes, pH 7.0 at a final protein concentration of 15–18 mg mL^−1^, according to [59]. After 15 min of incubation at 0 °C, the mixture was centrifuged at 15,000× *g* for 15 min. Then, the supernatant, supplemented with 1.0 mg of Cardiolipin, was applied to cold hydroxyapatite/celite (1:2) columns and eluted in the presence of the same Buffer A. As previously reported [49], the first fraction (1 mL) was collected and subsequently applied to a cold Matrex Gel Orange A column. Pure *Ht*DTC was isolated with an apparent molecular weight of 31.6 kDa [49]. All the operations were performed at 4 °C.

### 4.4. Reconstitution of the Rat OGC into Liposomes

Purified OGC was reconstituted into liposomes, as described previously [46]. Briefly, the reconstitution mixture was constituted of 200 µL of purified OGC, 100 µL of 10% (*w*/*v*) Triton X-114, 80 µL of 10% (*w*/*v*) egg yolk phospholipids in sonicated liposome form, 10 mM malate, 200 µL of 10 mg/mL Asolectin, and 10 mM Pipes, pH 7.0, in a final volume of 700 µL. After vortexing, to facilitate detergent removal by using the micro-batchwise method [60], the mixture was transferred to an Eppendorf tube (2 mL) containing 0.4 g Amberlite Bio-Beads SM-2 and rotated at 32 rpm. Finally, the proteoliposomes were recovered by gentle aspiration. All operations were performed at room temperature. The external substrate was removed by passing the formed proteoliposomes through a Sephadex G-75 column (0.7 × 15 cm), pre-equilibrated with 50 mM NaCl/10 mM Pipes (pH 7.0).

### 4.5. Reconstitution of the HtDTC into Liposomes

Purified *Ht-*DTC was reconstituted into liposomes as described previously [49]. Briefly, the reconstitution mixture was constituted of 200 µL of purified *Ht*DTC, 100 µL of 10% (*w*/*v*) Triton X-114, 100 µL of 10% (*w*/*v*) egg-yolk phospholipids in the form of sonicated liposomes; 10 mM 2-oxoglutarate; and 10 mM Pipes, pH 7.0, in a final volume of 700 µL. After vortexing, to facilitate detergent removal by the micro-batchwise method [60], the mixture was transferred to an Eppendorf tube (2 mL) containing 0.4 g Amberlite Bio-Beads SM-2 and rotated at 32 rpm. Finally, the proteoliposomes were recovered by gentle aspiration. All operations were performed at room temperature. The external substrate was removed by passing the formed proteoliposomes through a Sephadex G-75 column (0.7 × 15 cm), pre-equilibrated with 50 mM NaCl/10 mM Pipes (pH 7.0).

### 4.6. Transport Measurements

The first turbid fraction of proteoliposomes was collected from the Sephadex G-75 column, which is used for transport measurements using the inhibitor stop method [61]. The transport at 25 °C was started by adding 0.1 mM [^14^C] 2-oxoglutarate to proteoliposomes and, after 10 min, terminated by the addition of 350 mM pyridoxal 5′-phosphate (PLP). After incubation, the external substrate was removed by chromatography on Sephadex G-50 columns (0.7 cm diameter; 10 cm height) pre-equilibrated with 50 mM NaCl, and the radioactivity in the liposomes was measured. The experimental values were corrected by subtracting the respective control values. Km and Vmax values were determined by a computer-fitting program based on linear regression analysis.

### 4.7. Protein Quantification

The purified OGC carrier was identified by using SDS-polyacrylamide-gel electrophoresis of acetone-precipitated proteins in the presence of 0.1% SDS according to Laemmli method [62]. Staining was performed using the silver nitrate method [63]. The protein was determined by the Lowry method modified for the presence of non-ionic detergent [64]. All samples used for protein determination were subjected to acetone precipitation and dissolved in 1% (*w*/*v*) SDS.

### 4.8. Molecular Docking

The homology model of the OGC carrier was built and minimized according to criteria employed in previous works [37,38]. The nicotinamide adenine dinucleotide (NADPH) structures were fetched from the Protein Data Bank (entry code: NDP) and then energetically minimized using Ligprep tools available in the Schrödinger Suite [65].

The binding site for docking analysis was centered on the OGC cytosol facing region, including cytosolic loops between helices 2 and 3 (residues E107-T115) or helices 4 and 5 (residues K206-N218). The SP docking protocol used the OPLS4 force field [66]. 

### 4.9. Statistical Analysis

Statistical analyses were conducted using GraphPad Prism version 5.0 (GraphPad Software, San Diego, CA, USA). Data are presented as the mean ± standard deviation (S.D.) of a minimum of three independent experimental replicates. Data sets were subjected to a one-way analysis of variance (ANOVA) to detect significant differences among the groups. Dunnett’s post-hoc test was subsequently applied to perform multiple comparisons between each experimental condition and the control group. Statistical significance was set at a threshold of *p* < 0.05. Significance levels were denoted as follows: * *p* < 0.05, ** *p* < 0.01, and *** *p* < 0.001.

## 5. Conclusions

This study elucidates the significant modulatory effect of NADPH on the activity of the 2-oxoglutarate carrier (OGC) in mitochondria. We demonstrated that low concentrations of NADPH (0.010 mM) significantly enhance OGC transport activity by increasing the transport rate (Vmax) and slightly reducing the Km for 2-oxoglutarate, indicating a facilitated substrate entry into a more accessible OGC binding site. This modulation seems to arise from NADPH binding to the cytosolic loops of OGC. Supporting this hypothesis, molecular docking analyses revealed specific interactions between NADPH and key residues on these cytosolic loops, suggesting the presence of a potential regulatory binding site. Our findings further indicate that this modulatory effect of NADPH is unique to mammalian OGC, as it was not observed in the plant homolog (DTC) or other structurally/functionally related mitochondrial carriers. These results suggest that OGC may function as a sensor responsive to changes in redox states, potentially playing a crucial role in maintaining redox homeostasis and cellular metabolism under both physiological and pathological conditions. This work advances our understanding of mitochondrial function and highlights the complex regulation of mitochondrial carriers by redox states, opening new avenues for future research into mitochondrial oxidative stress responses and the development of therapeutic strategies for related diseases. 

## Figures and Tables

**Table 1:** The transport rate of reconstituted OGC in the presence of internal substrates ^#^.

Internal Substrate 10 mM	Transport Rate ± SD (nmol 10 min^−1^ mg^−1^ Protein)
Malate	3385 ± 120
None (Cl^−^ present)	21 ± 3 ***
GSH	24 ± 9 ***
GSSG	98 ± 9 ***
NADPH	70 ± 8 ***
NADP^+^	58 ± 1 ***

^#^ OGC proteoliposomes were preloaded with 10 mM of the indicated substrates using malate as a control. The transport was started by adding 0.1 mM [^14^C] 2-oxoglutarate for 10 min. The results are the means ± S.D. of at least three independent experiments. *p*-values were determined using a one-way ANOVA followed by Dunnett’s post-hoc test. *** indicates *p* < 0.001 compared to the control group.

**Table 2:** Inhibitory effect of GSH, GSSG, NADPH and NADP^+^ on the transport rate of reconstituted OGC ^##^.

External Inhibitor 2 mM	Transport Rate ± SD (nmol 10 min^−1^ mg^−1^ Protein)
CTRL	3384 ± 15
NADPH	4038 ± 80 ***
GSH	2998 ± 56 ***
NADP^+^	3138 ± 28 **
GSSG	3339 ± 35 ns

^##^ Proteoliposomes were preloaded with 10 mM malate and the transport was started by adding 0.1 mM [^14^C] 2-oxoglutarate. The inhibitors at a concentration of 2 mM were incubated with proteoliposomes for 3 min before adding the radiolabeled substrate. The control (CTRL) was represented by proteoliposomes exchanging 0.1 mM of external [^14^C] 2-oxoglutarate for the inner 10 mM malate, in the absence of any other external ligands (GSH, GSSG, NADPH and NADP^+^) replaced in the CTRL assays by the buffer (50 mM NaCl,10 mM Pipes, pH 7). The results were the means ± S.D. of at least three independent experiments. *p*-values were determined using a one-way ANOVA followed by Dunnett’s post-hoc test. Indicate ** *p* < 0.01, *** *p* < 0.001, ns *p* > 0.05 compared to the control group.

## Data Availability

The data supporting the findings of this study are available within the article. Raw data that support the findings of this study are available from the corresponding authors, upon request.
